# Temporal Lobe Cortical Thickness Correlations Differentiate the Migraine Brain from the Healthy Brain

**DOI:** 10.1371/journal.pone.0116687

**Published:** 2015-02-13

**Authors:** Todd J. Schwedt, Visar Berisha, Catherine D. Chong

**Affiliations:** 1 Mayo Clinic, Department of Neurology, Phoenix, Arizona, United States of America; 2 Department of Electrical Engineering, Arizona State University, Tempe, Arizona, United States of America; 3 Department of Speech and Hearing Science, Arizona State University, Tempe, Arizona, United States of America; National Scientific and Technical Research Council (CONICET)., ARGENTINA

## Abstract

**Background:**

Interregional cortical thickness correlations reflect underlying brain structural connectivity and functional connectivity. A few prior studies have shown that migraine is associated with atypical cortical brain structure and atypical functional connectivity amongst cortical regions that participate in sensory processing. However, the specific brain regions that most accurately differentiate the migraine brain from the healthy brain have yet to be determined. The aim of this study was to identify the brain regions that comprised interregional cortical thickness correlations that most differed between migraineurs and healthy controls.

**Methods:**

This was a cross-sectional brain magnetic resonance imaging (MRI) investigation of 64 adults with migraine and 39 healthy control subjects recruited from tertiary-care medical centers and their surrounding communities. All subjects underwent structural brain MRI imaging on a 3T scanner. Cortical thickness was determined for 70 brain regions that cover the cerebral cortex and cortical thickness correlations amongst these regions were calculated. Cortical thickness correlations that best differentiated groups of six migraineurs from controls and vice versa were identified.

**Results:**

A model containing 15 interregional cortical thickness correlations differentiated groups of migraineurs from healthy controls with high accuracy. The right temporal pole was involved in 13 of the 15 interregional correlations while the right middle temporal cortex was involved in the other two.

**Conclusions:**

A model consisting of 15 interregional cortical thickness correlations accurately differentiates the brains of small groups of migraineurs from those of healthy controls. Correlations with the right temporal pole were highly represented in this classifier, suggesting that this region plays an important role in migraine pathophysiology.

## Introduction

Migraine is a complex disorder manifesting with headaches and hypersensitivities to auditory, visual, olfactory, and somatosensory stimuli. [1, 2, 3, 4] Consistent with these migraine manifestations, existing studies suggest atypical structure, functional connectivity, and stimulus-induced activations of brain regions that participate in different aspects of pain processing, processing of visual, auditory, and olfactory stimuli, and regions participating in multisensory integration.[5, 6, 7, 8, 9, 10, 11, 12] In this study, we further investigated the migraine brain architecture by comparing interregional cortical thickness correlations in migraineurs to healthy control subjects. Brain interregional cortical thickness correlations represent underlying structural and functional connectivity amongst regions of the brain cortex. [13, 14, 15, 16]

This study investigated interregional cortical thickness correlations in migraineurs and healthy controls and developed a multivariable model of the correlations that most accurately differentiate the brains of small groups of migraineurs from those of small groups of healthy controls.

## Methods

### Subjects

Following Washington University School of Medicine and Mayo Clinic Institutional Review Board Approvals, 64 adult migraine subjects and 39 adult healthy control subjects provided written informed consent and were enrolled into this study. Migraine diagnoses were made according to the diagnostic criteria of the International Classification of Headache Disorders, 2^nd^ edition (ICHD-2). [17] Potential subjects were excluded if they had neurologic disease other than migraine, if they had abnormal brain MRI scans, or if they had contraindications to MRI. All subjects were screened for anxiety and depression using the State-Trait Anxiety Inventory and the Beck Depression Inventory. Control subjects could not have headaches other than the occasional tension-type headache (i.e. frequency had to be less than 3 headaches per month).

### MRI Acquisition Parameters

Subjects were imaged on one of two Siemens (Erlangen, Germany) MRI machines, each at a different institution: 1) MAGNETOM Trio 3T scanner using a 12-channel head matrix coil; or 2) MAGNETOM Skyra 3T scanner using a 12-channel head matrix coil. Structural scans included a high-resolution 3D T1-weighted sagittal magnetization-prepared rapid gradient echo (MP-RAGE) series (Trio parameters: TE = 3.16 ms, TR = 2.4 s, 1x1x1 mm voxels, 256x256 mm field of view (FOV), acquisition matrix 256 x 256; Skyra parameters: TE = 3.03 ms; TR = 2.4 s; 1x1x1.3 mm voxels; 256x256 mm FOV, acquisition matrix 256 x 256) and T2-weighted images in axial plane (Trio parameters: TE = 88 ms, TR = 6280 ms, 1x1x4 mm voxels, 256x256 mm FOV, acquisition matrix 256 x 256; Skyra parameters: TE = 84 ms; TR = 6800 ms; 1x1x4 mm voxels; 256x256mm FOV, acquisition matrix 256 x 256).

### Cortical Reconstruction and Segmentation

T1 MP-RAGE sequence image processing was performed using the FreeSurfer image analysis suite (version 5.3, http://surfer.nmr.mgh.harvard.edu/). To avoid postprocessing irregularities between workstations, all image post-processing was conducted using a single Mac workstation running OS X Lion 10.7.5 software. The methodology for this procedure is described in detail in prior papers [18, 19, 20, 21, 22, 23, 24, 25, 26, 27, 28, 29]. In short, processing includes skull stripping [29], automated Talairach transformation, segmentation of subcortical white matter and gray matter structures [22, 26] intensity normalization [30], tessellation of brain boundaries, automated topology correction [29], and surface deformation [18, 19, 20].

FreeSurfer output was visually inspected for errors before subject data were included for further analysis. Cortical thickness was defined as the distance from the boundary of the gray matter and white matter to the boundary of the gray matter and cerebral spinal fluid at each vertex along the brain surface [20]. Mean cortical thickness estimates for the automated parcellations of 35 regions over the left hemisphere and 35 regions over the right hemisphere were extracted from within FreeSurfer and exported to MATLAB (2007a, MathWorks) for further analysis.

### Statistical Analysis

Subject demographics, anxiety scores, and depression scores were compared between cohorts using independent sample t-tests or Fisher’s Exact tests as appropriate. Interregional cortical thickness correlations amongst the 70 brain regions that cover the cerebral cortex were calculated for all subjects.

To identify differences in cortical thickness correlations between migraineurs and controls, a pairwise cortical thickness correlation matrix was computed for each subject cohort. Euclidean distance was used to compare differences in correlations between the two subject groups. The 15 correlations yielding the largest Euclidean difference between the two subject cohorts were selected as features that differentiate the migraine brain from the healthy control brain. Subsets of six subjects from the same subject cohort (i.e. six migraine subjects, six control subjects) were randomly selected. The 15 cortical thickness correlations were computed for each group of six individuals. The Henze-Penrose divergence (HPD) measure was then used to quantify differences in the cortical thickness correlations between migraineurs and controls. [31] HPD values range from 0.5 (i.e. subject groups cannot be separated) to 1 (i.e. subject groups are completely separable).

To confirm the results obtained using the HPD measure, a classifier was trained and evaluated on the data under two conditions. In the first condition (Experiment 1), two data matrices (one for migraine and one for controls) were formed by randomly sampling groups of six subjects from each subject cohort (migraineurs or controls). This was performed for 500 different subsets of 6 from each subject cohort and, for each subset of 6, the 15 pairwise correlations features were computed. The data were concatenated into a single matrix, resulting in a matrix of 1000 samples and 15 features (i.e. interregional cortical thickness correlations). This concatenated matrix was then randomly split into a training set (700 subject subsets of six) and a test set (300 subject subsets of six). In the second condition (Experiment 2), we constrain our sampling criteria such that subjects that appear in the generation of the training set do not appear in the generation of the test set. This ensures that there is no overlap between the two training sets (e.g. if subjects 4 and 5 are used to generate the correlation features in the training set, those two subjects are excluded from the test set). Random subsets of 40 (of the 64 migraineurs) and 24 (of the 39 healthy controls) subjects were used to generate the training data and the remaining groups were used to generate the test data. As in Experiment 1, the training data consists of 700 subsets of 6 subjects and the test data consists of 300 subsets of 6 subjects (the two sets are sampled from non-overlapping subject sets). For both conditions, a classifier was trained (sparsity-constrained linear classifier) on the training set and then evaluated on the test set [32]. Multi-fold cross validation using data from the training set was used to set the parameter of the penalty constant in the sparsity-constrained linear classifier. The results are presented in terms of the Receiver Operating Curve (ROC) with bootstrapping used to evaluate the confidence bounds (100 bootstrapping replicates).

## Results

Data from 64 migraine subjects and 39 healthy control subjects were available for this analysis. Subject characteristics are illustrated in Table 1. There were no differences between migraineurs and controls for age, sex, or handedness. Although migraineurs had higher anxiety and depression scores, average scores for migraineurs and controls were within normal ranges, suggesting absence of anxiety and depression. Migraineurs averaged 11.2 days (+/- 8.5 days) with headache per month and 14.8 years (+/- 10 years) with migraine. Twenty-two migraineurs had chronic migraine while 42 had episodic migraine. Twenty-eight migraineurs had migraine with aura while 34 had migraine without aura (data missing from 2 subjects). Medications that could be considered migraine prophylactics were utilized by only 7 migraineurs.

**Table 1:** Subject Demographics and Migraine Characteristics.

	Migraine (n = 64)	Control (n = 39)	p-value
**Age** (years)	34.5 +/- 11.3	34.5 +/- 11	0.98
**Sex** (female/male)	51/13	29/10	0.63
**Handedness** (R/L)	59/5	33/6	0.32
**State Anxiety** (mean +/- SD)	27 +/- 6.8	24.5 +/- 5.4	.043
**Trait Anxiety** (mean +/- SD)	34.3 +/- 9.5	29.2 +/- 8.6	.006
**Depression** (mean +/- SD)	5.2 +/- 6.2	2.6 +/- 4.5	.015
**Aura** (yes/no) (n = 62)	28/34	N/A	N/A
**Headache days/month** (mean +/- SD)	11.2 +/- 8.5	N/A	N/A
**Years with migraine** (mean +/- SD)	14.8 +/- 10	N/A	N/A
**Migraine Prophylactic Medication** (yes/no)	7/57	N/A	N/A

State and trait anxiety scores were determined via the State-Trait Anxiety Inventory. Depression scores were determined via the Beck Depression Inventory II. Although anxiety and depression scores are higher in migraineurs than in controls, mean values are within normal ranges.

**Fig 1:**
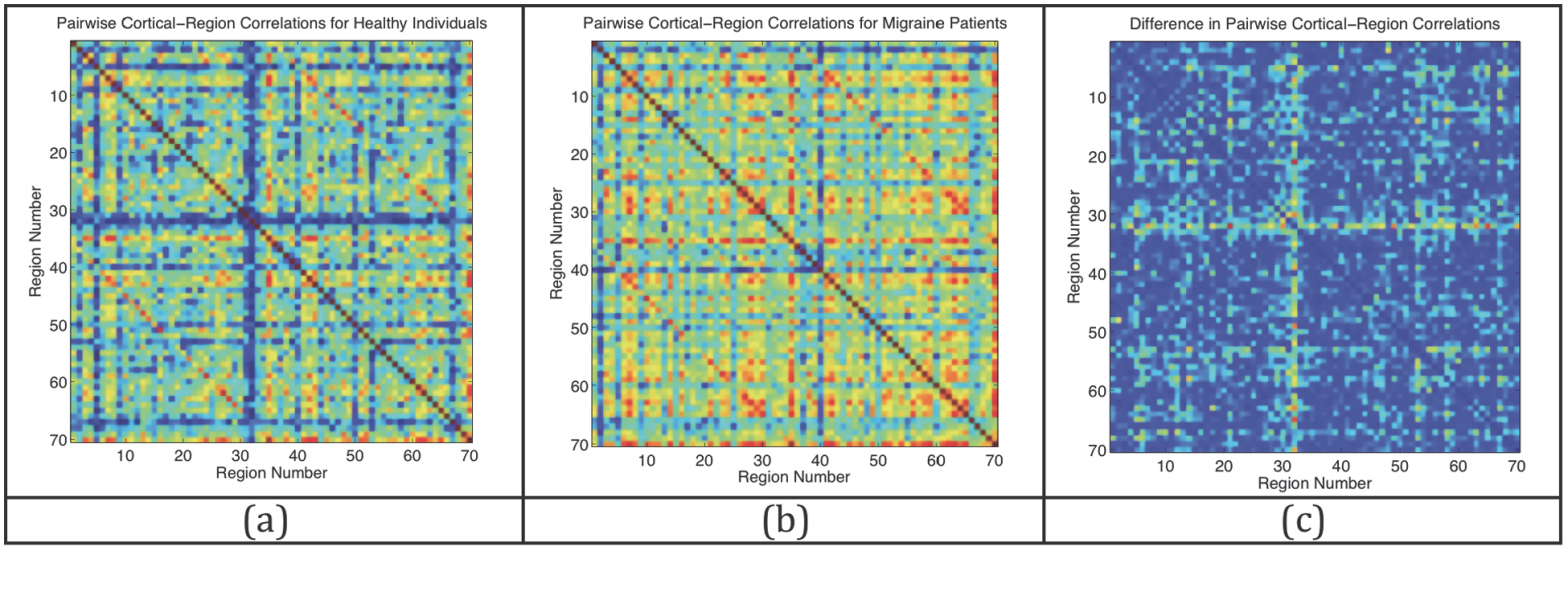
Regional Cortical Thickness Correlations. These matrices illustrate the interregional cortical thickness correlations amongst 70 brain regions that cover the cerebral cortex in: a) healthy controls, and b) migraine subjects. Panel (c) illustrates the differences in these correlations between migraine and control groups. In general, migraineurs had stronger correlations than controls, especially for the temporal pole correlations (feature 32). The color axis ranges from -0.1 (blue) to 1 (red).

Cortical thickness correlations amongst the 70 regions covering the cerebral cortex are illustrated in Fig. 1: panel (a) shows a plot of the pairwise interregional cortical thickness correlations for the healthy controls; panel (b) shows the same correlations for the migraine group; and panel (c) shows the absolute difference between the two correlation structures. Upon visual inspection of these matrices, it is apparent that there were differences in interregional cortical thickness correlation strengths between migraine and control subjects. In general, migraineurs had stronger cortical thickness correlations compared to controls who had weaker, sometimes negative, correlations. The correlation difference is especially pronounced for feature 32. This feature, which corresponds to the correlation between the cortical thickness of the temporal pole and other areas in the brain, was a key predictor for the migraine group.

**Fig 2:**
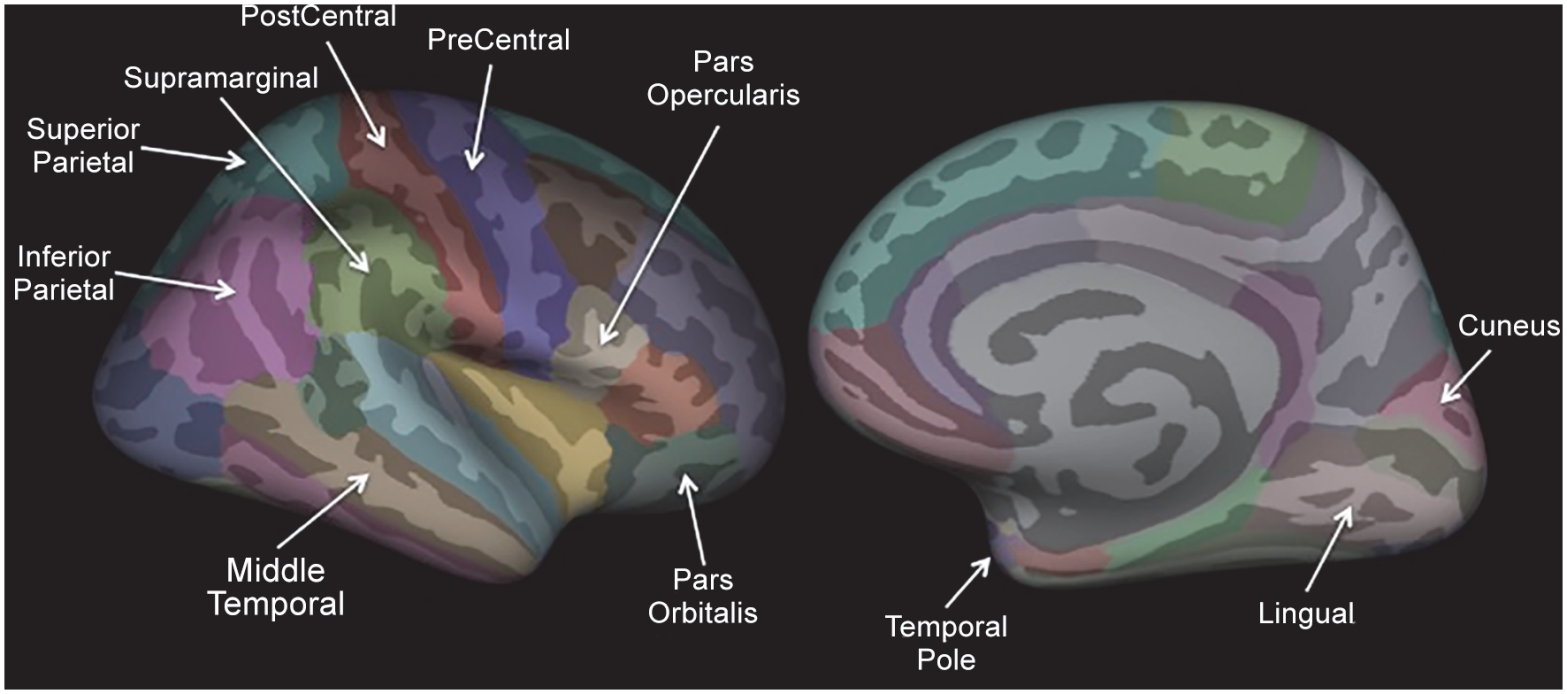
FreeSurfer Regions. The 35 cortical regions on the right hemisphere are demonstrated. Cortical regions that participated in the 15 interregional cortical thickness correlations that best differentiated migraineurs from controls are labeled. Although all regions are demonstrated on the right hemisphere, their actual laterality can be found in Fig. 3.

**Fig 3:**
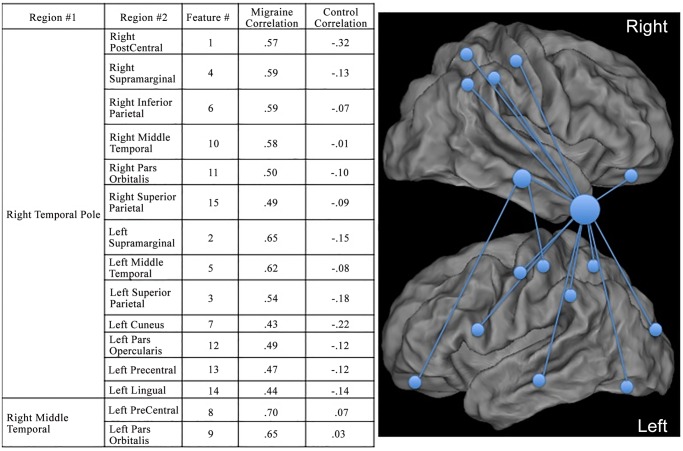
Interregional Cortical Thickness Correlations that Most Accurately Differentiate Migraineurs from Control Subjects. Fifteen interregional cortical thickness correlations most accurately differentiate migraineurs and controls. Locations of the cortical regions in these correlations are demonstrated with spheres placed on a 3-dimensional rendering of the brain. The size of the spheres is proportional to the number of interregional cortical thickness correlations that a region participates in. Thus, since the right temporal pole participates in 13 of the 15 correlations, it is represented by the largest sphere. Each of the 15 interregional cortical thickness correlations was assigned a “feature #”. The correlation strengths of the regions comprising each feature are reported for migraineurs and for healthy controls. For each of these features, migraineurs had stronger correlations than controls.

The 15 interregional cortical thickness correlations that most accurately classified subjects and the location of the regions comprising these correlations are illustrated in Figs. 2 and 3. Compared with controls, migraineurs had stronger cortical thickness intercorrelations with the right temporal pole and right middle temporal lobe.

**Fig 4:**
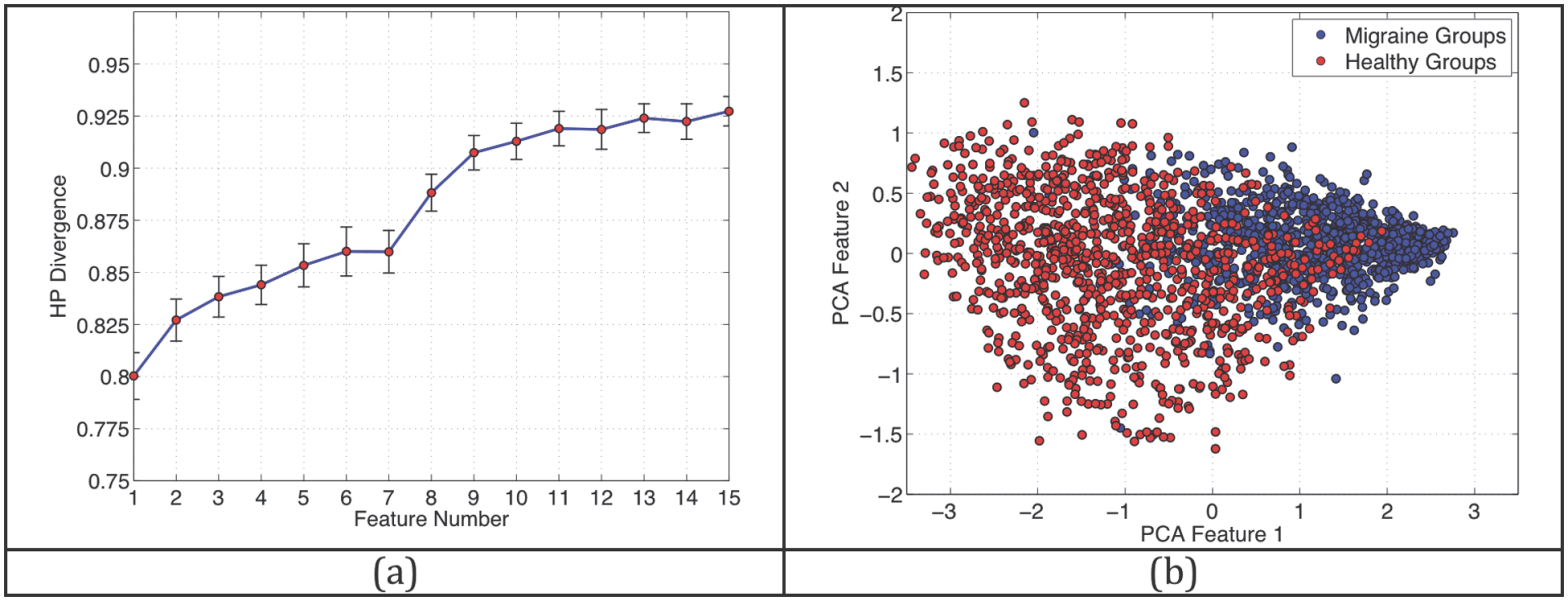
Divergence between Migraine and Controls. (a) The Henze-Penrose divergence between the healthy data and the migraine data showing that a classifier consisting of 15 interregional cortical thickness correlations separates subject cohorts at over 0.9. Values range from 0.5, meaning that subject groups cannot be separated, to 1, meaning that subject groups are completely separable. The regions comprising each feature (x-axis) can be found in Fig. 3. (b) A two-dimensional PCA embedding of data from the two groups illustrating the separability of migraineurs and controls.

**Fig 5:**
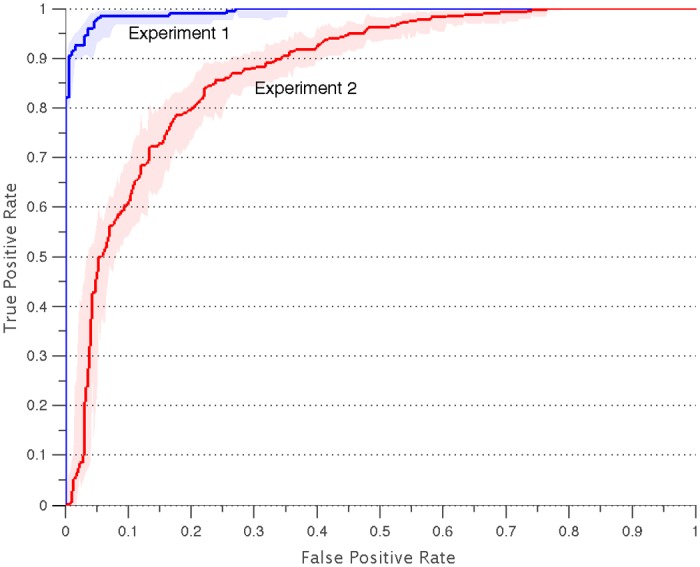
Receiver Operating Curve (ROC) for Classifiers in Experiments 1 and 2. The ROC curve showing the tradeoff between the True Positive Rate (sensitivity) and the False Positive Rate (1—specificity) for Experiments 1 and 2. Experiment 1 uses training and test data from the same group of patients and Experiment 2 uses training and test data from non-overlapping set of patients.

The Henze-Penrose divergence (HPD) measure was used to quantify differences in cortical thickness correlations between migraineurs and controls and serves as a proxy for classifier performance. [31], [33, 34] In panel (a) of Fig. 4, we plot the increase in HP divergence (or separability) as each additional feature from Fig. 3 is added to the set of features. This metric plateaus at over 0.9, implying that the classifier trained for separating between the two groups performs with a very high success rate. This is pictorially confirmed in panel (b), where we show a 2-dimensional embedding (using principal component analysis) of the 15-dimensional feature set for the two different groups. The separability between the healthy and the migraine subsets is readily apparent. To confirm the results obtained using the HPD measure, we trained and evaluated a classifier on the data, as described in the Methods statement. In Fig. 5, we show an ROC curve that shows the results for both experiments. Based on the tradeoff between the True Positive Rate and the False Positive Rate we see that the classifier in Experiment 1 significantly outperforms the classifier in Experiment 2. For example, for a fixed False Positive Rate of 0.1 (specificity of 0.9), the True Positive Rate (sensitivity) is 0.98 in Experiment 1 and 0.625 in Experiment 2. This is to be expected as the training and test data in the first experiment come from the same set of patients, whereas in experiment 2, the training and test data are generated from a non-overlapping set of patients. In either case, the results for both scenarios indicate significantly better-than-chance performance. The implication is that there exists a great deal of difference in the structural correlations between the two groups.

## Discussion

The main finding of this study is that interregional cortical thickness correlations with the right temporal pole and right middle temporal lobe are substantially different in migraineurs compared to healthy controls. Thirteen of fifteen interregional cortical thickness correlations that best differentiated the migraine brain from the brains of healthy control subjects involved the temporal pole, suggesting that this region is important for differentiating migraine brain structure from that of healthy controls and suggesting that the right temporal pole plays an important role in migraine pathophysiology.

Cortical thickness is a marker of gray matter integrity, reflective of the size, density and arrangements of cells.[14, 35] Cortical thickness correlations reflect anatomical connectivity between brain regions, with cortical thickness correlations being strong between regions that are axonally connected.[15, 36] Cortical thickness correlations are also thought to reflect functional connectivity, with functionally connected regions having strong cortical thickness correlations. [13, 14, 16, 37, 38, 39] Cortical thickness is not static, with changes occurring over time in association with aging, according to brain usage patterns, and in the presence of certain diseases.[35, 40, 41, 42] Likewise, covariations in cortical thickness amongst regions might result from shared insults, underlying genetic factors, and shared usage-driven plasticity.[14, 40, 43, 44, 45, 46, 47]

Diseases that are associated with atypical cortical thickness or atypical cortical thickness correlations include those typically considered as neurodegenerative disorders, such as Alzheimer’s disease, as well as disorders not classically considered neurodegenerative, such as acute and chronic pain.[14, 48] Atypical gray matter structure has previously been identified in patients with migraine.[49, 50, 51, 52, 53] However, investigations of cortical thickness correlations in patients with migraine are lacking from the literature. Contrasting cortical thickness correlations between subject groups is considered a valid method of comparing large-scale topological organization of the human cortex.[14, 15] Therefore, in this study we contrasted cortical thickness correlations in migraine to correlations in healthy controls in order to investigate the architecture of the migraine brain and to determine the cortical thickness correlations that most contribute to a multivariable model that differentiates migraineurs from controls.

Thirteen of the fifteen region pairs that best differentiated migraineurs and controls included the right temporal pole. This temporal pole region extends rostrally from the anterior portion of the temporal lobe caudally to the entorhinal cortex and from the medial aspect of the temporal lobe laterally to the superior or inferior temporal sulci.[54] The temporal pole participates in pain processing by mediating affective responses to painful stimuli and by acting as a multisensory integration zone responsible for processing painful, visual, auditory, and olfactory stimuli.[6, 7, 55] Several migraine neuroimaging studies have found the temporal pole to be hyperexcitable and to have stronger resting functional connectivity compared to healthy controls.[6, 7, 56, 57] Compared to non-migraineurs, interictal migraineurs have greater activation of the temporal pole in response to painful stimuli.[7] Temporal pole activation is further enhanced during a migraine attack compared to the interictal period and in migraineurs with more frequent migraine attacks compared to those with less frequent attacks.[7, 12, 58] Migraineurs have stronger functional connectivity of the temporal pole with other pain processing regions and other multisensory convergence zones including the temporoparietal junction, anterior cingulate cortex, insula, primary somatosensory cortex, spinal trigeminal nucleus, amygdala, caudate and pulvinar.[59, 60] Taken together, the findings of these previously published studies and our present study demonstrate the importance of the temporal pole in differentiating migraine brain structure from controls and suggest that the temporal pole plays an important role in migraine pathophysiology.

A limitation of the results reported herein is that the determination of the accuracy by which the model of interregional cortical thickness correlations differentiated the migraine brain from the brains of healthy controls was based on concurrent evaluation of groups of six migraineurs and six healthy controls, as opposed to using individual migraineurs. If the goal was to build a classifier that could potentially be used clinically for diagnosing migraine, the classifier would have to have the ability to differentiate the individual migraineur. Although that was not the goal of this study, we do theorize that a diagnostic classifier based upon MRI measures of brain structure could be a practical tool in clinical practice since the required MR sequences are collected during a routine clinical brain MRI, they do not take long to acquire, and they do not require contrast administration. Once ruling out secondary headache disorders, the diagnosis of migraine is determined according to a patient’s report of specific symptoms that they have during the migraine attack, as defined by the International Classification of Headache Disorders diagnostic criteria.[61] Although there are diagnostic tests that help to rule out secondary headache disorders, there are no tests used in the clinical setting that help to rule in a diagnosis of migraine. Although a diagnosis based upon symptoms is typically sufficient, there are situations when the migraine diagnosis cannot be made with certainty and availability of a diagnostic test would be of high utility: 1) differentiating migraine from other headache types that have similar symptoms, such as persistent post-traumatic headache and medication overuse headache; 2) assigning a migraine diagnosis when patients have difficulty or inability to report their symptoms; 3) to ensure a migraine diagnosis when enrolling a subject into a migraine research study. Although the use of two different MRI scanners and presence of clinical heterogeneity within our migraine group (e.g. varying headache frequencies, migraine with aura and migraine without aura) could be considered study limitations, we believe they represent strengths of this study. Nearly equal proportions of migraine and control subjects were imaged on each of the two MR scanners. Furthermore, prior multicenter studies of cortical thickness have found the use of different MR scanners to have negligible effects on study findings.[62] If the use of two MR scanners and the inclusion of a relatively heterogeneous group of migraine subjects had an effect on our study results, it would have reduced the accuracy of the classifier while making the results more generalizable. Finally, other more sophisticated feature selection algorithms could have been used to downselect from the total set of possible features (2485 total features from the correlation matrix) to 15 features. Two reasons motivated the initial down-selection to 15 features: (1) restricting our focus to only 15 features helps to reduce the effects of the curse of dimensionality since our sample size was relatively small compared to the full data dimension; and (2) the features contained regions that were consistent with other published studies in the literature.

## Conclusions

Interregional cortical thickness correlations of the right temporal pole and right middle temporal lobe differentiated the migraine brain from those of healthy controls. The right temporal pole was involved in 13 of 15 interregional cortical thickness correlations that most accurately differentiate the migraine brain, further emphasizing the likely importance of this region in migraine pathophysiology.
